# Evaluation of Pulsed-FRAP and Conventional-FRAP for Determination of Protein Mobility in Prokaryotic Cells

**DOI:** 10.1371/journal.pone.0025664

**Published:** 2011-09-28

**Authors:** Jacek T. Mika, Victor Krasnikov, Geert van den Bogaart, Foppe de Haan, Bert Poolman

**Affiliations:** Department of Biochemistry, Groningen Biomolecular Science and Biotechnology Institute, Netherlands Proteomics Centre and Zernike Institute for Advanced Materials, University of Groningen, Groningen, The Netherlands; J. Heyrovsky Institute of Physical Chemistry, Czech Republic

## Abstract

**Background:**

Macromolecule mobility is often quantified with Fluorescence Recovery After Photobleaching (FRAP). Throughout literature a wide range of diffusion coefficients for GFP in the cytoplasm of *Escherichia coli* (3 to 14 µm^2^/s) is reported using FRAP-based approaches. In this study, we have evaluated two of these methods: pulsed-FRAP and “conventional”-FRAP.

**Principal Findings:**

To address the question whether the apparent discrepancy in the diffusion data stems from methodological differences or biological variation, we have implemented and compared the two techniques on bacteria grown and handled in the same way. The GFP diffusion coefficients obtained under normal osmotic conditions and upon osmotic upshift were very similar for the different techniques.

**Conclusions:**

Our analyses indicate that the wide range of values reported for the diffusion coefficient of GFP in live cells are due to experimental conditions and/or biological variation rather than methodological differences.

## Introduction

In 1999 Elowitz and co workers [1] published a pioneering study on the mobility of proteins inside live *E. coli* cells, using Fluorescence Recovery After Photobleaching (FRAP). Although FRAP has been a well established tool for studying macromolecule mobility inside living cells since the 1970's [2], the attempts to use FRAP in bacteria have proven to be difficult mainly due to their small size. For example an *E.coli* cell is typically 1×3 µm, while human fibroblasts can easily reach dimensions over 100 µm. This property renders bacterial cells difficult to study as their dimension are only a few times larger than the diffraction limit of optical microscopy. Moreover, traditional FRAP protocols include a relatively large photo-bleaching spot and high laser intensity, both of which are not amenable for the small volume of bacteria.

Elowitz *et al.* [1] have modified the FRAP protocol by using a smaller bleaching spot and weaker laser power, so that sufficient non-photobleached GFP is left to measure fluorescence recovery. This approach has been successful implemented by others [3], [4], [5], [6]. Subsequently, other methods have been tailored to probe macromolecule diffusion in bacteria, including FRAP related techniques like pulsed–FRAP [7] and continuous photobleaching, using total internal reflection microscopy [8], [9], single molecule tracking [10] and fluorescence correlation spectroscopy [11]. For an overview of those techniques, we refer to Mika and Poolman [12].

There are now several studies that report the mobility of GFP and related proteins like YFP and mEos2, yielding diffusion coefficients for these proteins in the *E.coli* cytoplasm that range from 3 µm^2^/s [7], [13] through 6–7 µm^2^/s [1], [6] up to 14 µm^2^/s [5], [10]. The question arises whether this relatively wide range of values reflects differences in the methods used or biological and/or experimental variations?

We have now compared ‘conventional FRAP’ as initially used by Elowitz [1] and pulsed-FRAP, developed in our laboratory [7], to determine the diffusion of GFP in *E. coli* under well-defined conditions. We demonstrate that both techniques yield very similar distributions of diffusion coefficients of GFP in the cytoplasm under normal and osmotic stress conditions. We conclude that the different *D* values reported for GFP(-like) proteins in the literature [1], [5], [6], [7], [10], [13], [14] are a result of different handling of the cells and true biological variations rather than differences in the FRAP methods used.

## Materials and Methods

### Strains, growth and preparation of bacterial cells for microscopy

*Escherichia coli* K-12 strain MG1655 harboring pGFPCR [7] was grown as described previously [13]. Briefly, the cells were grown from single colonies in Luria Broth (10 g/L Bacto Tryptone (Becton Dickinson), 5 g/L Yeast extract (Becton Dickinson) plus 10 g/L NaCl (Merck)) supplemented with 100 µg/mL ampicillin (Sigma) at 37°C with vigorous shaking until the culture had reached an OD_600_ of 0.3–0.4. Leaky expression of GFP from the pGFPCR plasmid was sufficiently high to allow measurements and thus no inducer was added to the medium. Prior to the microscopy, the cells were washed twice with the NaPGCl medium (NaPGCl = 95 mM sodium phosphate, pH 7.0, 50 mM glucose plus 125 mM sodium chloride), which has an osmolality equal to that of LB (ΔLBOsm = 0) and very low fluorescence. For measurements the cells were either kept in NaPGCl or osmotically upshifted by supplementing the medium with additional NaCl. The osmolality of all solutions was measured by determination of their freezing point (Osmomat 030, Gonotec). For microscopy, 2 µl of cells was placed on poly-L-lysine (1% w/v) coated cover slips and measurements were carried out immediately. Each sample was imaged for periods no longer than 25 min. For each osmotic condition, a minimum of 20 single cells was analyzed. All measurements were performed at 20 +/− 1°C.

### Measurements of diffusion coefficients

Pulsed-FRAP measurements were carried out on a confocal microscope as described by van den Bogaart *et al.* [7]. Briefly, cells were first imaged with a confocal microscope and a low-intensity, diffraction limited laser beam was positioned in the middle of the cell. Subsequently, this laser beam was modulated using a shutter to apply short pulses, separated by time intervals without illumination to allow the fluorescence to recover. The fluorescent signal recorded during the pulses is influenced by the photobleaching of the GFP in the focal spot (decrease of fluorescence intensity) and by diffusion of the non-photobleached fluorophore into the focal spot (increase of fluorescence intensity). The fluorescence is linearly proportional to the concentration of GFP. The measured traces can be analysed using the Fick's second law where the GFP concentration fluctuations inside the cell *C(r, t):*

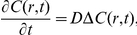



where *C* is the concentration of GFP, *Δ* is the LaPlace operator and *r* and *t* define the position and time point, respectively. We assume that the photo-bleaching rate is proportional to the intensity of the focused laser beam *I(r)* and thus obtain a bleaching constant *B*: 
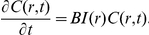



Finally, to obtain a diffusion coefficient (*D*), the traces are fitted numerically to a 2D diffusion model as described in detail by van den Bogaart *et al.* [7].

**Figure 1:**
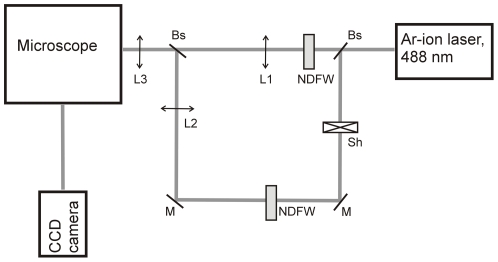
Optical scheme of the set-up used to perform conventional FRAP. Bs, beam splitter; M, mirror; NDFW, neutral density filter wheel; L1, L2, L3 lenses; Sh,shutter.

“Conventional FRAP” measurements were performed using an inverted microscope Observer D1 (Carl Zeiss, Jena, Germany) equipped with a Zeiss C-Apochromat infinity-corrected 1.2 NA 63× water immersion objective and a motorized X-Y translating stage for fine positioning of the cells. The optical part of the set-up is depicted in Fig. 1 and is very similar to the one reported by Konopka and co-workers [6]. The laser beam (488 nm, argon ion laser, Melles Griot, Carlsbad, CA, USA) was split into two beams. The first beam was focused to a diameter of around 1 µm and was used for photobleaching. The second beam (‘wide’) had a diameter of around 100 µm in the image plane and was employed to monitor the fluorescence recovery. The fluorescence emission was collected through the same objective and separated from the excitation beam by a dichroic mirror (Chroma Technology, Rockingham, VT, USA) and further directed through a 488 nm notch filter (CVI, Melles Griot, Carlsbad, CA, USA). The fluorescence signal was collected by a Cool-Snap HQ2 CCD camera (Photometrics, Tucson, AZ, USA).

**Figure 2:**
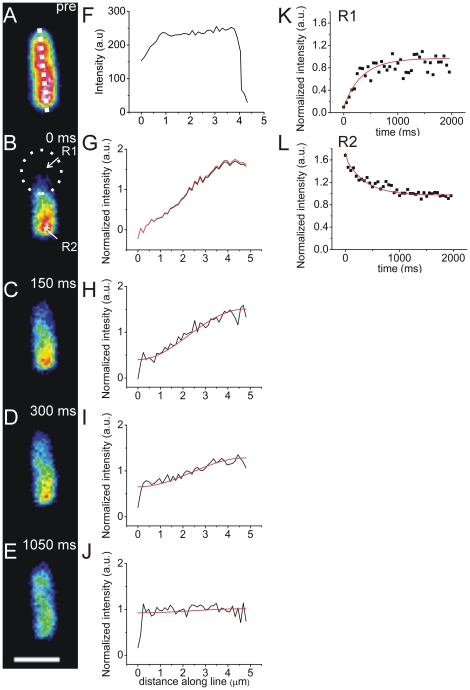
The ”conventional” FRAP method. A–E: snapshots of a cell during data acquisition; A: the cell before photobleaching (panel labeled “pre”); B–E: the cell during recovery after photobleaching (timestamp indicates the time after the photobleaching pulse). Dotted circle indicates the bleaching spot (B). Scale bar 2 µm. F–J: the fluorescence intensity along the dotted cross-section at given time points (corresponding to images on the left: A–E), where black is the normalized fluorescence intensity and red is the fit. K,L: the change of intensity in the course of the recovery at (K) the bleached pole of the cell (R1) and at (L) the opposite pole of the cell (R2). Black squares show the normalized data points and red lines the corresponding fits.

The cells were measured as described by Elowitz [1] and Konopka [6]. To position the desired bacterium in the focal plane of the microscope and in the bleaching area, bright-field transmittance microscopy mode was used. A fluorescence microscopy image of the cell before photobleaching was recorded (Fig. 2A). Subsequently, an area at the pole of the bacterium was photobleached (Fig. 2B) with a short (100 ms) focused light pulse. Immediately afterwards typically 40 images of the recovery of fluorescence were collected, using fluorescence illumination with the ‘wide’ beam. The frame rate to monitor the fluorescence recovery was adjusted to the speed of diffusion, i.e. every 25–50 ms for fast diffusion (normal osmotic conditions) and every 250 ms for severe osmotic upshift conditions. The analysis is described by Kumar *et al.* [4]. The images, recorded during the recovery phase, were analyzed numerically, using home written software as schematically depicted in Fig. 2G–J. A line was drawn through the longest axis of the bacterium and fluorescence intensity distributions along this cross-section (X-axis) were extracted from the image (Fig. 2B–J). To take into account intrinsic inhomogeneity of the fluorescence intensity along the X-axis, the ‘pre-photobleached’ distribution (Fig. 2F) was used to normalize all measured distributions. To obtain a diffusion coefficient, the software simulated the normalized fluorescence intensity distributions along the given cross-section during recovery (Fig. 2G–J). Following Elowitz [1], the one–dimensional diffusion approximation was assumed: 
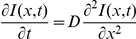
where *I* is fluorescence intensity and *D* is the diffusion coefficient; with boundary conditions: 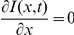
at the bacterial poles, corresponding to zero flux of GFP through the cell membrane. For final renormalization the distribution of GFP prior to photobleaching (Fig. 2F) was used. For the cell shown in Fig. 2, we obtained a diffusion coefficient of 6 µm^2^/s.

To illustrate the evolution of the fluorescence recovery, ‘single-spot’ fluorescence traces at positions R1 and R2 are depicted in Fig. 2K–L. using the classical approach introduced by Axelrod [2]:, these fluorescent traces (Fig. 2K–L), yielded a diffusion coefficient *D* of 1.5 µm^2^/s, assuming a beam diameter of 1 µm.

For each experimental condition, at least 20 individual cells were analyzed. The obtained diffusion coefficient values were plotted as histograms.

## Results and Discussion

### Pulsed FRAP and “conventional FRAP” yield similar diffusion coefficients

**Figure 3:**
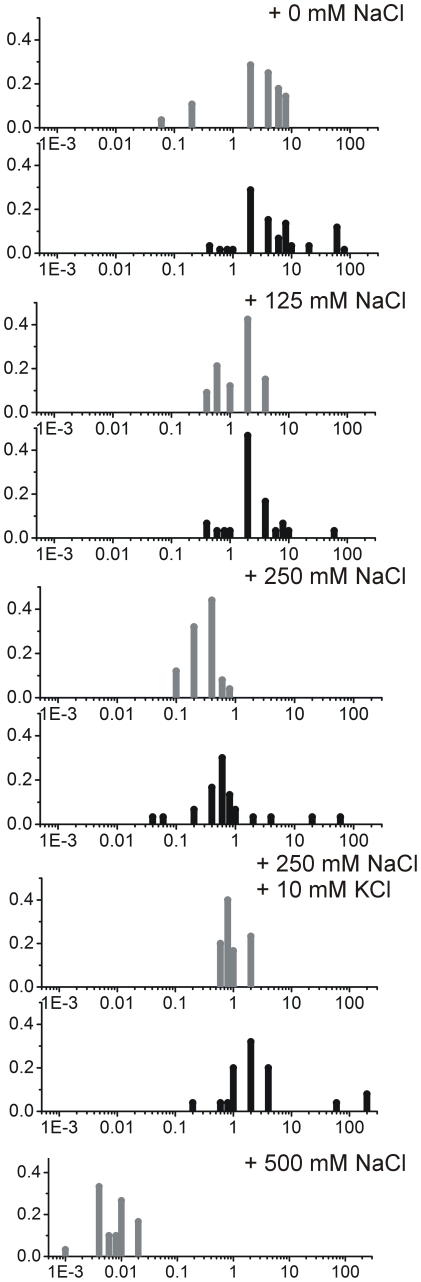
Comparison of pulsed-FRAP with “conventional FRAP”. Diffusion coefficients of GFP in the cytoplasm of *E. coli* measured with “conventional FRAP” (gray bars) and pulsed-FRAP (black bars) under normal osmotic conditions (top panel, “+ 0 mM NaCl”) and under conditions of osmotic upshift (lower panels, the extent of osmotic shock is indicated in the upper right corner of each panel). For the highest osmotic stress (lowest panel, “+500 mM NaCl”) only “conventional FRAP” data are presented. The number of cells measured in each experiment was at least 20.

Figure 3 presents histograms of distributions of diffusion coefficients of cytoplasmic GFP at different osmotic regimes. To ease the visual comparison of the two methods, the corresponding data from pulsed-FRAP and “conventional FRAP” have been plotted below each other. We note that for pulsed-FRAP measurements of very low molecule mobility are difficult to probe. Pulsed-FRAP requires that the time between photobleach light-pulses is long enough for complete recovery of the GFP in the cell. In severely stressed cells (500 mM NaCl or more) diffusion is very slow and the recovery is not homogenous. In fact, as reported previously above 500 mM of NaCl [7] one observes apparent barriers for diffusion. For these so-called plasmolyzing cells, pulsed-FRAP is not suitable to obtain quantitative information on protein mobility.

In general both pulsed- and “conventional”-FRAP give quite broad distributions of diffusion coefficients, which has been also observed by others [1], [6], [7], not only for GFP but also for other macromolecules [13]. The wide range of distributions is in part due to errors in the measurements and data analysis, but a large part is due to population heterogeneity. Pulsed-FRAP yields somewhat broader distributions of diffusion coefficients, which might be a result of the changes of the cell position in respect to the focal volume during the measurement.

Despite the difference in the methodologies, both techniques yield similar maxima in the distributions of values and the decrease in diffusion of GFP is proportional to the strength of the osmotic shock applied. Moreover, the diffusion coefficients of osmotically stressed cells increase in the presence of osmoprotectants (Fig. 3 panel “+250 mMNaCl+10 mM KCl”).

### Differences between methods

From a practical point of view “conventional FRAP” may be more suited to probe protein mobility in live cells than pulsed-FRAP. With “conventional FRAP” snapshots of the whole cell are recorded, which gives information about the distribution of the GFP inside the cytoplasm (i.e. whether it is homo- or heterogenous) and it can be easily observed if the cell moves during the measurement. In case of pulsed-FRAP the movement of cell cannot be observed until the end of the series of bleaching pulses, which could hamper data acquisition. Moreover, the data analysis on average takes only a few minutes with “conventional FRAP”, while it can take up to hours to calculate a diffusion coefficient with pulsed-FRAP.

On the other hand pulsed–FRAP maybe better suited for smaller cells or organelles. Pulsed-FRAP also proved suitable for measuring the mobility of very fast diffusing NBD-glucose, which has a poor photostability [13]. This would be technically challenging to perform with “conventional FRAP”. The cell length of *E.coli* is typically 3 µm. The average diffusion coefficient of NBD-glucose in the cytoplasm of *E.coli* under normal osmotic condition is close to 50 µm^2^/s, which means that after photobleaching the fluorescence can fully recover within less than 30 ms. Since this dye is not very bright and quite photolabile, fast CCD imaging is insufficient to capture good quality FRAP data.

### Why are the reported diffusion constants different?

In the recent literature, the diffusion coefficients for GFP in the *E.coli* cytoplasm, grown and analyzed under normal osmotic conditions, ranged from 3 to 14 µm^2^/s. This variation may be partly due to the methods of extracting the representative average values. Some authors take the arithmetic average [6], [7], [8]. Upon visual inspection of the data obtained for *E.coli* (Fig. 3), it is apparent that the median value may actually be a better approximation of the ‘average’ diffusion coefficient. This approach has been implemented in later studies [5], [13].

Another possible reason for the differences in the diffusion coefficients is the composition of the growth media. It can be observed that cells grown in rich media like LB show lower mobility of proteins [6], [7], [13] than cells grown in minimal media [5]. Particularly striking are the values reported by the group of Weisshaar [5], [6]. Using “conventional FRAP”, they report diffusion coefficient for GFP [6] of 6–7 µm^2^/s for LB grown cells, whereas cells grown in a minimal MOPS-based medium showed values of 14 µm^2^/s [5]. Moreover, they reported that the culture's history has a major impact on the mobility of GFP diffusion [5]. Cells adapted to high osmolality of growth tend to display a lower decrease in diffusion coefficients upon exposure to osmotic upshift than cells grown in media of normal osmolality.

The observation that in bacteria grown in rich media the diffusion of macromolecules is slower than in bacteria grown in minimal medium may have a plausible biological rationale. Bacteria grown in rich media divide more often and synthesize more mRNA [15] and have a higher number of ribosomes [16] as compared to bacteria growing and dividing more slowly in minimal medium. In this context the overall macromolecular crowding (often thought to be the main cause for slower diffusion in the cytoplasm as compared to dilute aqueous solutions) may be rather constant, however, the nature of the crowding agent would change. It is tempting to speculate that in the cytoplasm of bacteria grown on rich media the diffusion is slower due to a higher abundance of ribosomes that are bulkier crowders (11.4 nm radius, [5]) than average cytoplasmatic proteins (2–3nm diameter). Moreover, the increased content of mRNA, which forms long unfolded chains, may additionally hinder macromolecule diffusion. The hypothesis that not only the cytoplasm crowding itself, but also the nature of the crowders contributes to slowed diffusion remains yet to be proven experimentally.

### Conclusions

We have shown that, under a variety of osmotic conditions, pulsed-FRAP and “conventional-FRAP” yield very similar diffusion coefficients of GFP in the cytoplasm of *E.coli*. We speculate that the different values reported in the literature are due to variations in the constitution of the cells, i.e. as determined by their growth media and history.
